# Identification of putative chemosensory receptor genes from yellow peach moth *Conogethes punctiferalis* (Guenée) antennae transcriptome

**DOI:** 10.1038/srep32636

**Published:** 2016-09-23

**Authors:** Xing Ge, Tiantao Zhang, Zhenying Wang, Kanglai He, Shuxiong Bai

**Affiliations:** 1 State Key Laboratory for Biology of Plant Disease and Insect Pest, Institute of Plant Protection, Chinese Academy of Agricultural Science, Beijing 100193, China

## Abstract

The yellow peach moth, *Conogethes punctiferalis*, is an extremely important polyphagous insect in Asia. The chemosensory systems of moth play an important role in detecting food, oviposition sites and mate attraction. Several antennal chemosensory receptors are involved in odor detection. Our study aims to identify chemosensory receptor genes for potential applications in behavioral responses of yellow peach moth. By transcriptomic analysis of male and female antennae, 83 candidate chemosensory receptors, including 62 odorant receptors, 11 ionotropic receptors and 10 gustatory receptors were identified. Through Blast and sequence alignment, the highly conserved co-receptor Orco was annotated, eight unigenes clustered into pheromone receptors, and two clustered as sugar receptor. Among the IRs, one unigenes was similar with co-receptors IR25a. Expression levels of 50 odorant receptors were further evaluated by quantitative real-time PCR in antennae. All the ORs tested were detected in antennae and some of which were associated with sex-biased expression. The chemosensory receptors identified in *C. punctiferalis* provide a foundational resource for further analysis on olfaction for behavior. The expression profiles of ORs in antennae indicated variant functions in olfactory recognition, and our results provided the possibility for the potential application of semiochemical to control this pest moth.

Insect olfactory system enables insects to detect and discriminate different odor molecules from their environment. The odorant and pheromone perception in insects are a complex series of process that transfer external stimulus from the environment to behavioral response. Diverse proteins consisting in this signal transduction pathway include odorant binding proteins (OBPs), chemosensory proteins (CSPs), chemosensory receptor (CRs), odorant degrading enzymes (ODEs) and sensory neuron membrane proteins (SNMPs)^1^,^2^,^3^,^4^,^5^,^6^,^7^.

The gene family encoding CRs in insects mainly divides into three groups: olfactory receptors (ORs), gustatory receptors (GRs) and ionotropic receptors (IRs)^8^,^9^,^10^,^11^,^12^. The insect ORs and the GRs were firstly identified in the genomic analysis of *Drosophila melanogaster*^13^. Previous studies suggested that the OR and GR families belong to a superfamily based on the transmembrane domain^13^,^14^. Both ORs and GRs are seven transmembrane-domain receptors with an inverted transmembrane topology in contrasts to the OR of mammalian^14^,^15^,^16^. ORs are housed targeted in the dendritic membrane of olfactory sensory neurons (OSNs). Most OSNs express a single OR gene, along with odorant receptor co-receptor (Orco)^17^. Insect OR genes are highly diverse, and their numbers among species are various^13^,^18^,^19^,^20^,^21^,^22^. ORs have been shown to form a voltage-gated ion channel following heterodimerization with Orco, which is an ortholog of the *D. melanogaster* OR, DmOr83b^23^,^24^. ORs in Lepidopteran pheromone system are classified into two major groups, pheromone receptors (PRs) and general odorant receptors according to perception of chemicals^25^. PRs are thought to be associated with olfactory signal transduction of pheromone compounds in peripheral olfactory reception, and general odorant receptors are considered to detect plant volatiles. PRs were reported in several species^20^,^26^,^27^,^28^. Also pheromone-pheromone receptor relationships also have been identified. In *Bombyx mori*, general odorant receptor BmOR19 responds to linalool, furthermore, BmOR45 and BmOR47 respond to benzoic acid, 2-phenylethanol, and benzaldehyde respectively^29^. While PRs (BmOR1 and BmOR3) respond specifically to bombykol (E10Z12-16: OH) and bombykal (E10Z12-16: Ald) respectively, which are major and minor sex pheromone components in *B. mori*.^30^,^31^. Although we classified receptors on the basis of their responses to chemicals, their functions are still unknown. PRs are more conserved compared with other ORs^32^,^33^. Orco is the only highly conserved OR protein among divergent insect species, which suggests the crucial role in chemical recognition^34^,^35^. The GRs are generally expressed in gustatory receptor neurons (GRNs) within gustatory organs. Differently, several GRs are usually expressed in one neuron. Most GRs sense non-volatile compounds, including sugar and bitter compounds^36^. Interestingly, Some GRs expressed in antennae cannot identify volatile odorants, but respond to carbon dioxide^37^. IRs belongs to a variant subfamily of ionotropic glutamate receptors (iGluRs), involving in detection of chemical signals^38^,^39^. Based on the expression location, two subfamily of IRs are identified: conserved antennal IRs and divergent IRs, indicating the function of IRs in olfactory and taste^39^. In antennal IRs, IR8a and IR25a appear to act as co-receptors and form heteromeric complexes with odor- specific receptors^40^,^41^. In contrast with ORs, many IR-expressing neurons expressed two or three IR genes^17^.

The yellow peach moth *Conogethes punctiferalis* (Guenée) (Lepidoptera: Crambidae), is a serious polyphagous insect of crop (sunflower, maize, sorghum, cotton, etc.) and fruit (peach, plum, durian, etc.). It’s hard to control by traditional method, due to larvae bored the inside fruit or stem^42^,^43^. Few olfactory receptor genes have been reported in this moth^44^, however more receptor genes investigated allows a better understanding of the molecular basis of olfaction. Identification of the chemosensory genes can lead to comprehensive understanding of how to recognize and locate host for this moth. In the present study, the antennal transcriptome of *C*. *punctiferalis* were sequenced on the Illumina HiSeq 4000 platform. In total 83 chemoreceptor genes including 62 ORs, 11 IRs and 10 GRs were identified. The expression levels of ORs, which may play important roles in chemoreception of *C*. *punctiferalis* were analyzed by using of quantitative real-time PCR (qRT-PCR). The results assist with the identification of genes involved in *C. punctiferalis* olfactory, help better understand the gene expression in sex, and provide the molecular basis for further study of pheromone and host volatile recognition.

## Results

### Illumina sequencing

To identify the chemosensory protein genes from *C. punctiferalis*, the cDNA from male and female antennae were sequenced using the Illumina HiSeqTM 4000 sequencing platform. A total of 59,375,582 and 57,353,054 raw reads were obtained from female and male antennae, respectively. After removing adaptor sequences, low quality sequences and containing N sequences, 58,053,354 and 55,592280 clean reads were generated from the antennae of female and male raw data, respectively. After assembled, the two datasets result in 112,933 contigs with a mean length of 902 bp and an N50 of 1750 bp. From these datasets, 76,486 unigenes were obtained with a mean length of 727 bp and an N50 of 1499 bp (Table 1). The raw reads of the *C. punctiferalis* are available from the SRA database (accession number: (SRX 1604701 and SRX 1604705).

### Functional annotation of assembled unigenes

**Figure 1:**
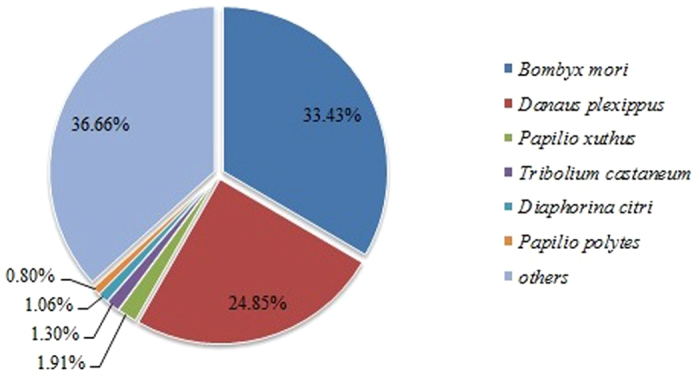
Best hits of the BLASTx results.

**Figure 2:**
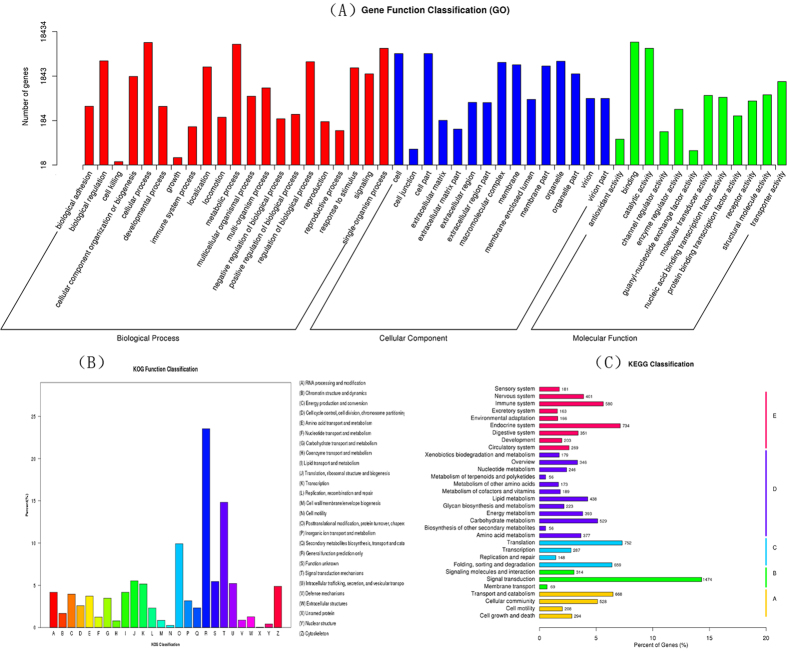
(**A**) Gene Ontology (GO) analysis for the transcriptomic sequences. (**B**) euKaryotic Ortholog Groups (KOG) annotation for the unigenes.(**C**) KEGG pathway annotation of the transcriptome. (**A**) Cellular processes. (**B**) Environmental information processing. (**C**) Genetic information processing. (**D**) Metabolism. (**E**) Organismal systems.

The proportion of unigenes annotated in at least one database of Nr, Nt, KO, SwissProt, PFAM, GO, KOG reach to 40%. Only 4.75% unigenes were annotated in all databases (Table 2). Using BlastX database, 26,114 (34.14%) unigenes were matched to known proteins. For species distribution, the results showed 33.43%, 24.85%, and 1.91% of protein similar with the sequences from *B. mori*, *Danaus plexippus* and *Papilio xuthus*, respectively (Fig. 1). GO annotation was used to classify the function of transcripts according to the GO terms (Fig. 2A). In the biological process terms, cellular, metabolic and single-organism were the highest classified. In the cellular component terms, cell, cell part and organelle were the most abundant. In the molecular function category, the genes expressed in the antennae were mostly related to binding, catalytic activity and transporter activity. KOG annotation was based on the relationship between orthologous genes from different species (Fig. 2B). Using KOG functional annotation, “General function prediction only”, “Signal transduction”, and “Posttranslational modification, protein turnover, chaperones” were three largest groups assigned to. For KEGG annotation, 10,298 unigenes were classified into five groups, Cellular processes, environmental information processing, genetic information processing, metabolism and organismal systems (Fig. 2C). Transport and catabolism, signal transduction, translation, carbohydrate metabolism, endocrine systems were the main pathways in each group, respectively.

### Putative chemoreceptor genes

All the unigenes were searched by BLAST to identified chemoreceptor genes. Total 62 OR genes, 11 IR genes and 10 GR genes were identified through bioinformatics analysis. All the chemosensory receptor genes of *C. punctiferalis* were submitted to the GenBank (accession numbers: KX084452-KX084521 and KX096203-KX096215). Of these genes, 45 unigenes (39 ORs and 6 IRs) showed sequence identity to the previously identified in *C. punctiferalis* which are already submitted in GenBank. Among the identified ORs, 38 were found to represent full-length open reading frames (ORF) with 4–7 transmembrane domains (Table 3). Half of those sequences were annotated against homologs of *Ostrinia furnacalis*. As expected, Orco/OR2 was identified with high identity compared with the conserved insect OR2/Orco. The IRs and GRs sequences in *C*. *punctiferalis* antennal transcriptome were identified according to the similarity with known insect IRs and GRs. Seven of eleven putative IRs were similar with IRs of *O*. *furnacalis*. Six unigenes with full-length ORF, contained 3~4 transmembrane domains. Of GR genes, 3 putative GR genes contained full-length ORF with 6 transmembrane regions, while 7 sequences were partial (Table 4).

### Phylogenetic tree

**Figure 3:**
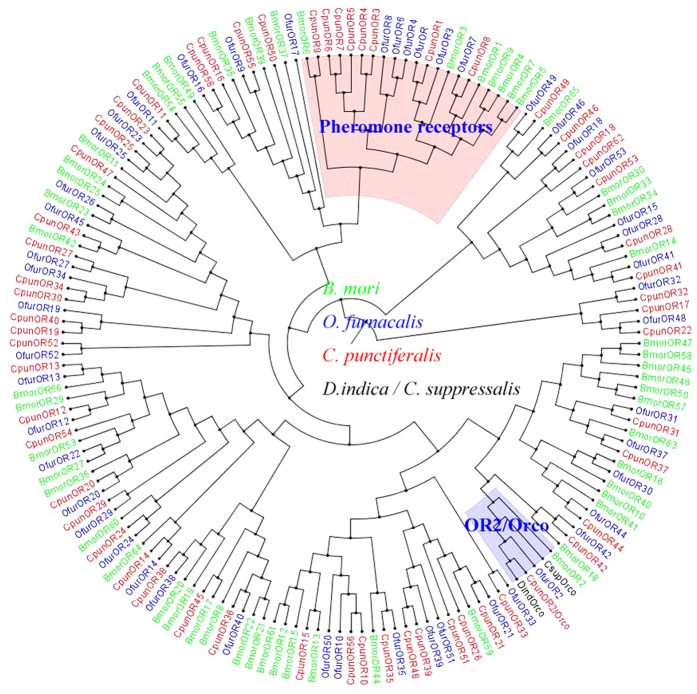
Phylogenetic relationship of putative olfactory receptors from *Conogethes punctiferalis* and other insects. The tree was constructed by MEGA 5.2 program using the neighbor-joining method with the Bootstrapping model by 1000 replication.

**Figure 4:**
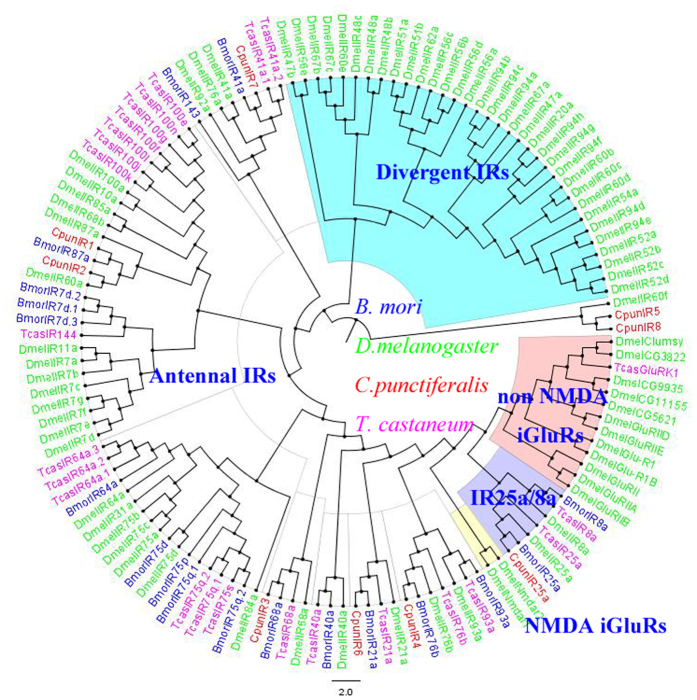
Phylogenetic relationship of putative ionotropic receptors from *Conogethes punctiferalis* and other insects. The tree was constructed by MEGA 5.2 program using the neighbor-joining method with the Bootstrapping model by 1000 replication.

**Figure 5:**
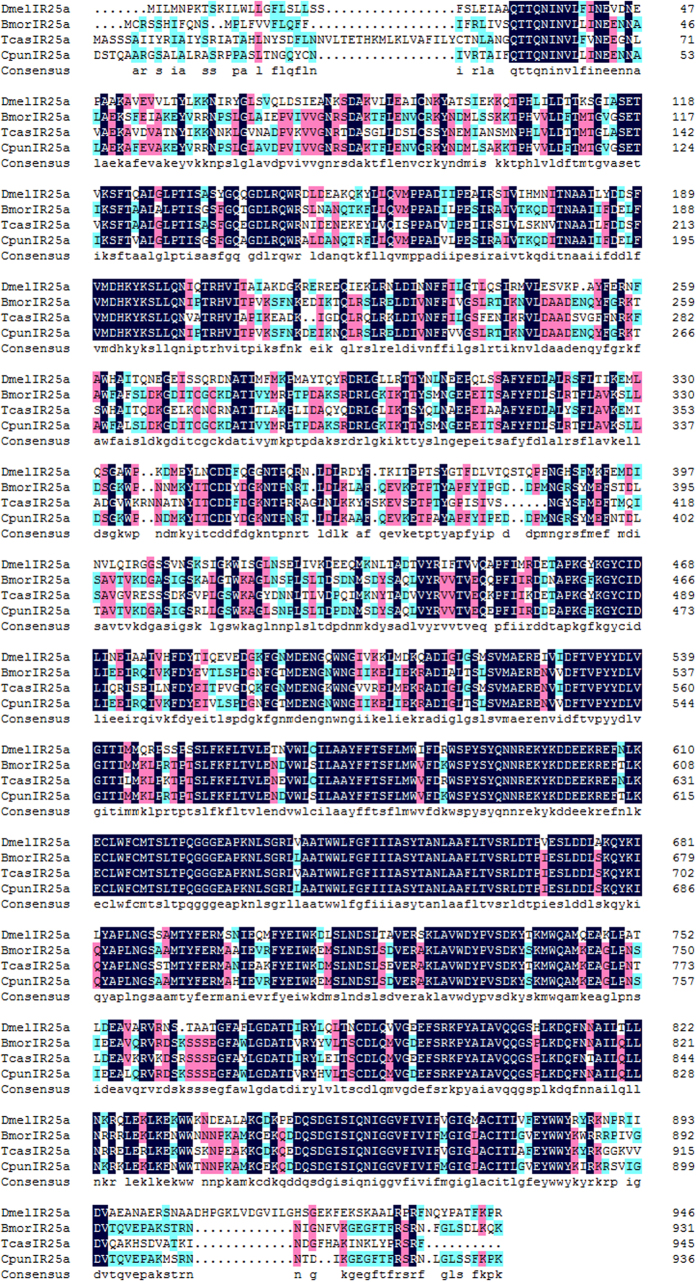
Amino acid alignment of the putative IR25a with other insects. Blue shadings indicate the same sequence among insects. Pink shading indicated amino acids which show 75% identity between sequences. Green shading ink shading indicated amino acids which show 50% identity between sequences.

**Figure 6:**
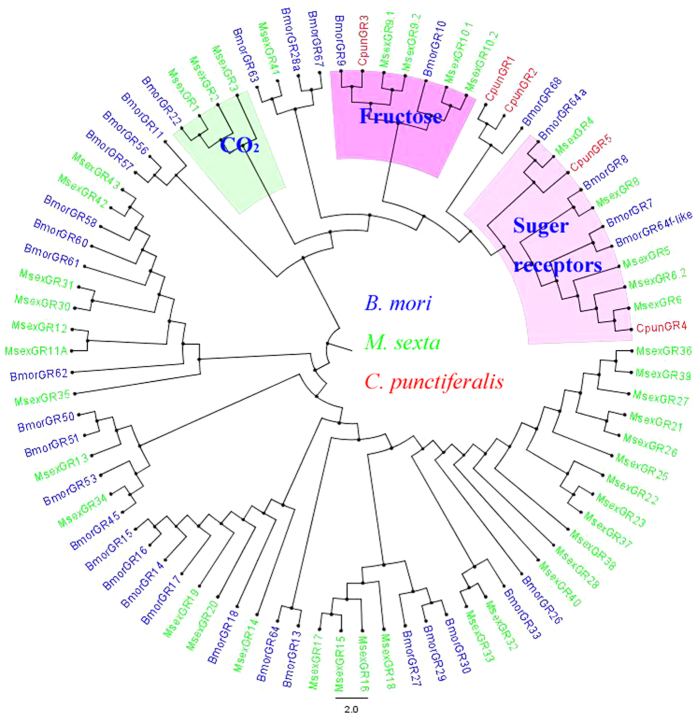
Phylogenetic relationship of putative gustatory receptors from *Conogethes punctiferalis* and other insect. The tree was constructed by MEGA 5.2 program using the neighbor-joining method with the Bootstrapping model by 1000 replication.

Phylogenetic tree of ORs was constructed using the sequences of 162 ORs from *B. mori*, *O. furnacalis* and *C. punctiferalis* (Fig. 3). The hits with a length of less than 200 amino acids were ignored. The OR sequences were clustered into PRs, Orco and other divergent ORs. The 8 amino acid sequences, OR1 and OR3-9 clustered into a clade with pheromone receptors of *B*. *mori* and *O*. *furnacalis*. Orco/OR2 was highly conserved and clustered with Orco/OR2 family. In the neighbor-joining tree of IRs (Fig. 4), the IRs were clustered into ionotropic glutamate receptors (iGluRs), IR25/IR8a and other divergent IRs. Among the nine IRs in *C. punctiferalis*, CpunIR25a grouped with the highly conserved IR25a/IR8a. Alignment analysis revealed that the similarity of IR25a was higher than 76.3% in *D. melanogaster*, *B. mori*, *Tribolium castaneum* and *C. punctiferalis* (Fig. 5). CpunIR4, CpunIR6, CpunIR3, CpunIR7 shared identity with IR76b, IR21a, IR68a, IR41a from *D. melanogaster*, *B. mori* and *T. castaneum*, respectively. Unfortunately, we cannot obtain the sequences belong to iGluRs. In phylogenetic tree of the GRs, GRs from *B. mori*, *Manduca sexta* and *C. punctiferalis* were analyzed (Fig. 6), CpunGR5 and CpunGR4 were respectively classified into BmorGr64a and MexGR6 subgroup, which were known as sugar receptors. CpunGR3 was cluster into a clade of fructose receptors.

### Expression level of OR genes in female and male antennae

**Figure 7:**
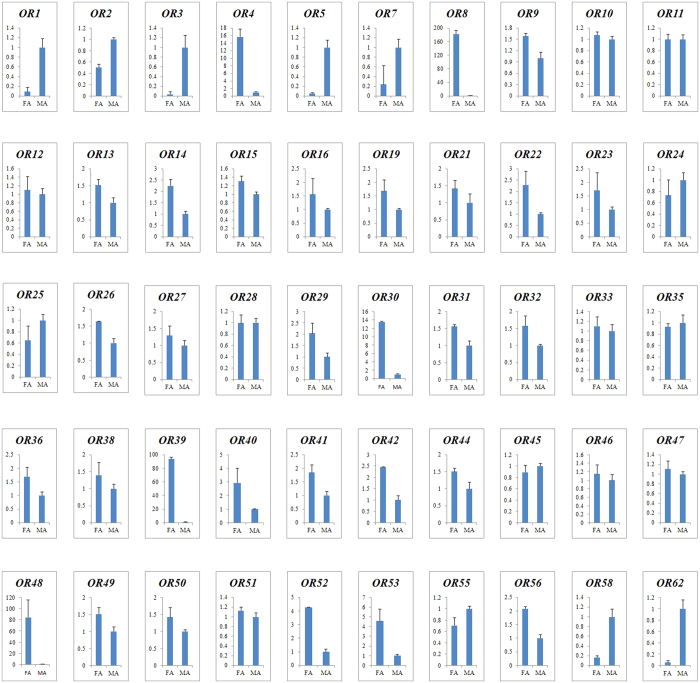
Relative expression levels of putative ORs in the female and male moth antennae. FA: female antennae; MA: female antennae. The expression levels were estimated using delta delta C_T_ method. Standard error for each sample is represented by error bar.

Quantitative real-time PCR (qPCR) was used to analyze the expression levels of 50 OR genes in the antennae of male and female moths. The results indicated that all the tested genes were detected in antennae. Among the OR genes, OR8, OR39, OR48, OR4 and OR30 were highly enriched in female, with 182.6, 93.9, 84.4, 15.6, and 13.5 times to male, respectively. PR subfamily members, OR1, OR3, OR5 and OR7 were highly expressed in male antennae, as well as the odorant receptor OR62 (Fig. 7). Meanwhile, Orco/OR2, OR26, and OR8 had high expression level in female with an FPKM value of 534.09, 50.52, and 43.43, respectively. Orco/OR2, OR3 and OR6 had high expression level in male with an FPKM value of 523.08, 283.65, 170.90, respectively (Table 3). As co-receptor, Orco/OR2 was most abundant in female and male antennae. Because OR4, OR39, OR48 and OR53 were only detected in female antenna, so we cannot obtain the FPKM value (Table 3).

## Discussion

With the rapidly development of next generation sequencing technology, a large number of unigenes were identified. RNA-seq effectively increased the depth of sequencing compared to the previous sequence data from cDNA library^46^,^47^,^48^. From the transcriptome of *C. punctiferalis*, we identified 83 chemoreceptors including 62 ORs, 11 IRs and 10 GRs. In Crambidae family insect, totally 56 ORs were identified in *O. furnacalis*^20^, and in other moths, Cao *et al*. found 47 ORs and 20 IRs in *Chilo suppressalis*^49^, suggested that the olfactory related genes we identified were equivalent to other moths. Total number of identified OR genes varied in different orders or species due to sequencing methods and depth, and/or sample preparations. In Lepidopteran, *B. mori*, totally 66 ORs were reported in genomic data^50^. In Dipteran, there were 79 candidate OR genes from *Anopheles gambiae* and in Hemipteran, 170 OR genes from *Apis mellifera* were annotated^18^,^21^. Especially in *Nasonia vitripennis*, total number of ORs was up to 301^51^. However, identified OR genes were only obtained from the antennae, ORs expressed in other tissues might be difficult to identify and the physiological states perhaps influence the amount of ORs in the antennae^22^. It seemed that more OR genes in Hymenopteran insect, especially the parasitoid wasp, might be due to need more receptors were needed to identify and locate the host.

In Lepidopteran insects, the PRs are more conserved and clustered in a subgroup within the OR family^9^. In our study, OR1 and OR3-9 of *C. punctiferalis* were clustered with pheromone receptors of *B. mori* and *O. furnacalis*, and we inferred that some or all of them might be putative pheromone receptors. But the expression profiles of these sequences showed that not all of them were male-specific. Koenig considered that at least one of the putative PR should exhibit male-specific expression associated with trichoid sensilla^9^. Recent studies showed that some PR genes expressed in both sexes. The PR1, 2 and 4 identified from the antennae of *C. suppressalis* have been detected in both male and female antennae^49^. From the antennal transcriptome of *Spodoptera littoralis*, four unigenes were enriched in male antennae, only two of which were annotated as putiative PRs and found to be expressed in antennae of both sexes^48^. In *B. mori*, Bmor19, Bmor45 and Bmor47 were also reported as female-specific or highly female-biased receptors, which responded to linalool, benzoic acid and benzaldehyde, suggesting the potential roles in detecting oviposition site and male-produced sex pheromone^29^. In our study, five unigenes were enriched in the female antennae than in the male, which may have provide important function during the host plant seeking.

IRs are derived from ionotropic glutamate receptors and comprise a novel family of chemosensory receptors. In *D. melanogaster*, 15 of total 66 IR genes were specifically expressed in antennae^38^ that was similar with our study that only 11 IRs were identified from the antennae transcriptomes of *C. punctiferalis*. Phylogenetic analysis showed that IRs from *C. punctiferalis* were not closely related to the iGluRs. Animal iGluRs are highly conserved family of ligand-gated ion channels^39^, while IRs are extremely divergent exclusive of IR8a and IR25a^38^. IR25a and IR8a are most similar primary sequence to iGluRs, suggesting that they are the IR genes most similar to the ancestral IRs^9^,^39^,^52^. In our study, CpunIR25a clustered with the highly conserved IR25a from *B. mori*, *D. melanogaster* and *T. castenum*, while in *N. vitripennis* and *Microplitis mediator*, two putative IR25a orthologues were identified^22^,^39^. Therefore, the congruent function of IR25a orthologues is still not clear. In Lepidoptera insect, IR8a were identified from the antennal transcriptome of *Helicoverpa armigera*, *H. assulta* and *C. suppressalis*^26^,^49^. Surprisingly, IR8a was not found in our antennal transcriptome. We speculated that it maybe need more deeply sequencing and increasing the samples, suggesting that IR8a maybe not expressed in our present samples.

In the previous studies, 56 and 77 candidate GR genes were respectively identified from the *Drosophila* and *Acyrthosiphon pisum* genome^14^,^53^. In *B. mori*, 65 GR genes were annotated from the silkworm genome^54^. However, only 10 GRs were identified from the antennal transcriptome of *C. punctiferalis*. It might be that GRs have a low expression level in the antennae and mainly expressed in gustatory organs (proboscis, legs, wings and genitalis)^55^,^56^. GRs have been divided into four classes: non-fructose sugar, bitter, CO_2_, and fructose receptors^9^,^54^. In phylogenetic tree of GRs, the gustatory receptor genes, GR3-5 were clustered into sugar and fructose receptors, suggesting the potential sugar detection function in the antennae of this moth. However, the recent report showed a wide range of non-gustatory sensory functions of GRs^57^, indicating that GRs may have more divergent functions in insect antennae.

In conclusion, the main purpose of this study was to identify the chemosensory receptor genes involved in the chemoreception. Based on the transcriptomic analysis, 83 CRs were identified from the antennae of *C. punctiferalis*. Our method was successful in identifying CR genes with low-expressing levels and the results made it possible for further studies on the molecular level and behaviors, providing the possibility to applicate potential target genes for controlling this pest.

## Materials and Methods

### Insects rearing and antennae collection

*C. punctiferalis* larvae were collected from the infested sunflower at Langfang Experimental Station of Chinese Academy of Agricultural Sciences, Hebei Province, China. In laboratory, the larvae were raised on fresh corn ear under conditions 27 ± 1 °C, with 70–80% relative humidity (RH) and a photo period of 16:8 h light: dark (L:D). After eclosion, adults were fed with 10% honey solution. The antennae (70 pairs of each sex) from adults within three days after eclosion were separately collected and frozen immediately in liquid nitrogen.

### RNA extraction

Total RNAs were separately extracted and purified using Trizol Reagent (Invitrogen, Carlsbad, CA, USA) following the product manuals. The integrity of total RNA was assessed with Agilent 2100 (Agilent Technologies Inc., CA, USA) and the purity was detected on a NanoDrop 2000 spectrophotometer (NanoDrop products, Wilmington, DE, USA). The concentration was accurately measured by Qubit. Illumina sequencing was performed at Novogen Co., Ltd. Beijing, China, The cDNA libraries were sequenced using Illumina HiSeq 4000 system under effective concentration. In order to obtain the high-quality transcriptome sequence data, the raw reads were filtered to eliminate adaptor sequences and low quality reads. Reads with uncertain nucleotides larger than 10% of the fragment sequence were removed. Trinity *de novo* program with a default k-mer was used to assemble the clean reads^45^. Redundant sequences were removed to obtain unigenes sequences by means of selecting longest transcript contigs.

### Unigenes annotation and classification

To obtain comprehensive information of gene functions, assembled unigenes were searched against Nr (NCBI non-redundant protein sequences), Nt (NCBI nucleotide), Pfam (Protein family), KOG (euKaryotic Ortholog Groups), SwissProt, KEGG (Kyoto Encyclopedia of Genes and Genomes), GO (Gene Ontology) database using BlASTX with an E-value < 10^−5^. CDS (coding sequence) were predicted in two-step process, the unigenes were firstly aligned using NR and SwissProt protein database to obtain ORF. If the sequences mismatch with the two databases, estscan (version 3.0.3) software was used to predict the ORF to obtain the nucleic acid and amino acid sequence. FPKM (fragments per kilobase pairs per million mapped reads) was used for gene expression analysis.

### Identification of chemosensory receptor genes

Homology searches of OR, GR and IR sequence were performed using BLAST (http://blast.ncbi.nlm.nih.gov/blast.cgi), and the ORFs were predicted using ORF finder (http://www.ncbi.nlm.nih.gov/gorf/gorf.html). Transmembrane domain was predicted using TMHMM (http://www.cbs.dtu.dk/services/TMHMM/). Phylogenetic tree was constructed by MEGA 5.2 software using the neighbor-joining method with the Bootstrapping model by 1000 replication. The evolutionary distances were computed by using the Poisson correction method. The phylogenetic tree image was further created by Figtree software.

### Quantitative real-time PCR

The expression levels of ORs were examined in male and female antennae using qRT-PCR. Total RNA was extracted as described above. First-strand cDNAs were synthetize followed One-Step gDNA removal and cDNA Synthesis kit (TransGen Biotech, China) with oligo dT-primer following the kit manual. The primers for qPCR were designed using Primer premier5.0 program (Table S1). qRT-PCR were performed on ABI 7500 fast real-time PCR system (Applied Biosysterm, USA) in a reaction volume of 20 μL using SYBR Premix Ex Taq II (Tli RNaseH Plus) master mix (Takara-Bio, Shiga, Japan), according to the manufacturers’ instructions. Two-step program were performed as follows, 95 °C for 30 s, followed by 40 cycles of 95 °C for 3 s and 60 °C for 30 s. For each gene, three biological replications were performed with each biological replication measured in three technique replications. Relative quantification was analyzed using the comparative 2^−ΔΔCT^ method, with the housekeeping genes *β-actin* (accession number JX119014) as the reference gene.

## Additional Information

**How to cite this article**: Ge, X. *et al*. Identification of putative chemosensory receptor genes from yellow peach moth *Conogethes punctiferalis* (Guenée) antennae transcriptome. *Sci. Rep.* **6**, 32636; doi: 10.1038/srep32636 (2016).

## Supplementary Material

Supplementary Information

## Figures and Tables

**Table 1:** Summary of assembled contigs and unigenes.

	Contigs	Unigenes
Total length (bp)	101,865,459	55,582,691
Minimum length (bp)	201	201
Mean length (bp)	902	727
Median length (bp)	411	320
Maximum length (bp)	29,150	29,150
N50 (bp)	1,782	1,499
N90 (bp)	306	254

**Table 2:** Summary of annotations of unigenes.

	Number of Unigenes	Percentage (%)
Annotated in NR	26,114	34.14
Annotated in NT	11,354	14.84
Annotated in KO	10,298	13.46
Annotated in SwissProt	18,229	23.83
Annotated in PFAM	18,142	23.71
Annotated in GO	18,434	24.1
Annotated in KOG	11,475	15
Annotated in all Databases	3,640	4.75
Annotated in at least one Database	30,596	40
Total Unigenes	76,486	100

**Table 3:** Odorant receptors in *Conogethes punctiferalis* antennae.

contigs	Gene name	Accession number	residues	Full length	Top blastx hit	Score	E-value	% ID	TM	FPKM	
										Female	Male
c45607_g1	OR1	KX084452	449	Yes	BAG71417.1 | olfactory receptor-1 [*Diaphania indica*]	739	0.00E + 00	81%	4	0.68	9.61
c50176_g1	OR2 /Orco	KX084453	474	Yes	AGF29886.1 | odorant co-receptor [*Conogethes punctiferalis*]	951	0.00E + 00	99%	7	534.09	523.08
c45303_g1	OR3	KX084454	329	No	AFK30395.1 | odorant receptor 3 [*Ostrinia furnacalis*]	280	3.00E−87	42%	4	2.33	283.65
c35425_g1	OR4	KX084455	418	No	BAJ22891.1 | odorant receptor [*Ostrinia furnacalis*]	283	5.00E−87	39%	3	11.5	0
c43578_g2	OR5	KX084456	421	Yes	NP_001296037.1 | odorant receptor 13a-like [*Plutella xylostella*]	318	1.00E−100	45%	4	3.86	91.95
c43248_g1	OR6	KX084457	419	No	AFK30403.1 | odorant receptor 6 [*Ostrinia furnacalis*]	316	1.00E−99	43%	4	0.17	170.9
c42117_g1	OR7	KX084458	378	Yes	AIZ94614.1 | putative pheromone receptor OR6 [*Ostrinia nubilalis*]	175	2.00E−47	41%	7	0.08	24.79
c40235_g2	OR8	KX084459	428	Yes	BAG71424.1 | olfactory receptor [*Diaphania indica*]	449	1.00E−151	50%	6	43.43	9.77
c39383_g1	OR9	KX084460	426	Yes	AFC91723.1 | putative odorant receptor OR15 [*Cydia pomonella*]	211	3.00E−61	45%	6	5.81	1.27
c50460_g1	OR10	KX084461	394	Yes	BAR43452.1 | putative olfactory receptor 10 [*Ostrinia furnacalis*]	475	1.00E−162	59%	6	5.04	2.8
c40543_g1	OR11	KX084462	389	Yes	BAR43453.1 | putative olfactory receptor 11 [*Ostrinia furnacalis*]	493	4.00E−170	68%	6	5.17	4.22
c44553_g2	OR12	KX084463	353	No	BAR43454.1 | putative olfactory receptor 12 [*Ostrinia furnacalis*]	505	3.00E−175	65%	4	31.85	12.76
c46066_g1	OR13	KX084464	402	Yes	BAR43455.1 | putative olfactory receptor 13 [*Ostrinia furnacalis*]	645	0.00E + 00	81%	5	32.09	14.17
c46413_g1	OR14	KX084465	416	Yes	BAR43456.1 | putative olfactory receptor 14 [*Ostrinia furnacalis*]	546	0.00E + 00	64%	6	19.59	8.43
c50375_g1	OR15	KX084466	388	Yes	NP_001166603.1 | olfactory receptor 13 [*Bombyx mori*]	442	3.00E−150	61%	6	22.17	9.13
c46668_g4	OR16	KX084467	420	Yes	BAR43458.1 | putative olfactory receptor 16 [*Ostrinia furnacalis*]	523	1.00E−180	60%	5	10.49	4.84
c47537_g1	OR17	KX084468	418	No	EHJ78030.1 | olfactory receptor 29 [*Danaus plexippus*]	574	0.00E + 00	73%	0	2.79	2.99
c45438_g1	OR18	KX084469	424	No	BAR43460.1 | putative olfactory receptor 18 [*Ostrinia furnacalis*]	593	0.00E + 00	74%	6	5.9	3.44
c45263_g1	OR19	KX084470	415	Yes	BAR43461.1 | putative olfactory receptor 19 [*Ostrinia furnacalis*]	290	6.00E−90	40%	7	13.51	7.2
c42557_g3	OR20	KX084471	399	Yes	BAR43462.1 | putative olfactory receptor 20 [*Ostrinia furnacalis*]	437	1.00E−147	59%	5	24.84	8.24
c42436_g1	OR21	KX084472	395	Yes	AGK90020.1 | olfactory receptor 17 [*Helicoverpa assulta*]	461	2.00E−157	64%	5	25.06	19.27
c14092_g1	OR22	KX096208	150	No	XP_013191807.1 | PREDICTED: gustatory and odorant receptor 22 [*Amyelois transitella*]	305	2.00E−99	94%	3	0.91	0.22
c45775_g1	OR23	KX084473	401	Yes	BAR43467.1 | putative olfactory receptor 25 [*Ostrinia furnacalis*]	423	5.00E−142	52%	6	14.52	7.73
c46864_g1	OR24	KX084474	450	Yes	BAR43466.1 | putative olfactory receptor 24 [*Ostrinia furnacalis*]	627	0.00E + 00	80%	6	16.34	14.37
c49436_g1	OR25	KX084475	432	Yes	BAR43467.1 | putative olfactory receptor 25 [*Ostrinia furnacalis*]	671	0.00E + 00	78%	6	10.79	10.78
c44068_g1	OR26	KX084476	396	Yes	NP_001166611.1 | olfactory receptor 59 [*Bombyx mori*]	363	1.00E−118	47%	5	50.52	26.05
c42831_g1	OR27	KX084477	401	Yes	BAR43469.1 | putative olfactory receptor 27 [*Ostrinia furnacalis*]	647	0.00E + 00	86%	6	16.3	6.65
c43774_g1	OR28	KX084478	326	No	BAR43470.1 | putative olfactory receptor 28 [*Ostrinia furnacalis*]	309	2.00E−98	47%	6	22.87	14.79
c38000_g1	OR29	KX084479	413	Yes	AJF23799.1 | olfactory receptor OR32 [*Planotortrix octo*]	365	8.00E−119	48%	5	7.71	2.3
c47502_g1	OR30	KX084480	458	Yes	AIG51850.1 | odorant receptor [*Helicoverpa armigera*]	177	5.00E−50	72%	6	21.24	0.9
c45926_g1	OR31	KX084481	388	Yes	CUQ99414.1 | Olfactory receptor 34 [*Manduca sexta*]	399	4.00E−133	55%	7	12.74	7.83
c45783_g1	OR32	KX084482	390	Yes	AII01045.1 | odorant receptor [*Dendrolimus houi*]	354	2.00E−115	44%	6	4.86	1.1
c46810_g1	OR33	KX084483	404	No	BAR43475.1 | putative olfactory receptor 33 [*Ostrinia furnacalis*]	646	0.00E + 00	81%	7	4.97	1.95
c47522_g3	OR34	KX084484	306	No	BAR43476.1 | putative olfactory receptor 34 [*Ostrinia furnacalis*]	320	6.00E−103	56%	4	0.69	14.2
c41738_g1	OR35	KX084485	430	Yes	BAR43477.1 | putative olfactory receptor 35 [*Ostrinia furnacalis*]	809	0.00E + 00	89%	6	3.52	1.89
c42979_g1	OR36	KX084486	328	No	BAR43478.1 | putative olfactory receptor 36 [*Ostrinia furnacalis*]	370	1.00E−122	60%	6	13.92	5.12
c49487_g1	OR37	KX084487	389	Yes	BAR43479.1 | putative olfactory receptor 37 [*Ostrinia furnacalis*]	579	0.00E + 00	77%	6	11.94	5.43
c50585_g1	OR38	KX084488	398	Yes	BAR43480.1 | putative olfactory receptor 38 [*Ostrinia furnacalis*]	411	2.00E−137	49%	6	10.58	7.68
c42903_g1	OR39	KX084489	405	Yes	BAR43481.1 | putative olfactory receptor 39 [*Ostrinia furnacalis*]	249	2.00E−74	35%	5	13.98	0
c46193_g1	OR40	KX084490	414	No	BAR43461.1 | putative olfactory receptor 19 [*Ostrinia furnacalis*]	296	6.00E−92	44%	5	20.14	6.25
c45009_g1	OR41	KX084491	356	Yes	BAR43483.1 | putative olfactory receptor 41 [*Ostrinia furnacalis*]	429	5.00E−145	61%	6	4.75	1.93
c40483_g1	OR42	KX084492	370	No	BAR43484.1 | putative olfactory receptor 42 [*Ostrinia furnacalis*]	452	6.00E−155	61%	6	4.26	1.55
c44512_g1	OR43	KX084493	326	No	NP_001091818.1 | olfactory receptor 42 [*Bombyx mori*]	305	4.00E−97	54%	6	10.81	6.51
c45800_g1	OR44	KX084494	433	Yes	BAR43486.1 | putative olfactory receptor 44 [*Ostrinia furnacalis*]	555	0.00E + 00	69%	5	28.41	14.73
c44781_g1	OR45	KX084495	401	Yes	NP_001157210.1 | olfactory receptor 17 [*Bombyx mori*]	333	5.00E−107	41%	7	13.46	8.28
c45498_g3	OR46	KX084496	215	No	BAR43488.1 | putative olfactory receptor 46 [*Ostrinia furnacalis*]	377	6.00E−127	83%	2	9.53	5.48
c39174_g1	OR47	KX084497	203	No	XP_013195066.1 | PREDICTED: putative odorant receptor 92a [*Amyelois transitella*]	272	2.00E−86	63%	2	1.94	1.09
c28776_g1	OR48	KX084498	277	No	BAR43481.1 | putative olfactory receptor 39 [*Ostrinia furnacalis*]	199	3.00E−57	39%	4	1.45	0
c42096_g2	OR49	KX084499	431	Yes	BAR43491.1 | putative olfactory receptor 49 [*Ostrinia furnacalis*]	523	0.00E + 00	66%	6	14.61	7.74
c43755_g1	OR50	KX084500	410	Yes	KOB74670.1 | Odorant receptor 50 [*Operophtera brumata*]	477	6.00E−163	53%	7	6.07	1.65
c47178_g1	OR51	KX084501	330	No	AII01110.1 | odorant receptor [*Dendrolimus kikuchii*]	393	2.00E−131	53%	4	9.2	4.48
c30858_g1	OR52	KX084502	408	Yes	BAR43494.1 | putative olfactory receptor 52 [*Ostrinia furnacalis*]	491	1.00E−168	56%	6	7.22	1.26
c40944_g1	OR53	KX084503	402	Yes	BAR43495.1 | putative olfactory receptor 53 [*Ostrinia furnacalis*]	496	1.00E−170	66%	5	15.15	0
c48055_g1	OR54	KX084504	410	Yes	AFC91736.1 | putative odorant receptor OR28 [*Cydia pomonella*]	457	4.00E−155	52%	5	14.89	5.16
c49083_g1	OR55	KX084505	415	Yes	BAR43458.1 | putative olfactory receptor 16 [*Ostrinia furnacalis*]	538	0.00E + 00	63%	5	32.58	30.96
c48693_g1	OR56	KX084506	393	Yes	BAR43452.1 | putative olfactory receptor 10 [*Ostrinia furnacalis*]	425	4.00E−143	56%	6	20.86	8.35
c45498_g2	OR57	KX084507	215	No	BAR43488.1|putative olfactory receptor 46 [*Ostrinia furnacalis*]	340	2.00E−112	75%	3	10.91	4.52
c16587_g1	OR58	KX096203	195	No	BAR43458.1 | putative olfactory receptor 16 [*Ostrinia furnacalis*]	234	3.00E−71	61%	2	0.69	0.78
c38597_g3	OR59	KX096204	125	No	ACF32962.1 | olfactory receptor 4 [*Helicoverpa armigera*]	229	1.00E−70	87%	1	1.62	1.12
c10513_g1	OR60	KX096205	107	No	BAR43458.1 | putative olfactory receptor 16 [*Ostrinia furnacalis*]	156	4.00E−43	70%	2	0.4	1.46
c84526_g1	OR61	KX096206	107	No	AIT69908.1 | olfactory receptor 66 [*Ctenopseustis herana*]	122	1.00E−30	51%	1	1.65	0.98
c47522_g1	OR62	KX096207	135	No	XP_013165286.1 | PREDICTED: odorant receptor 85b-like [*Papilio xuthus*]	102	7.00E−23	35%	0	0.69	14.2

**Table 4:** Ionotropic and gustatory receptors in *Conogethes punctiferalis* antennae.

Contigs	Gene name	Accession number	Residues	Full length	Top blastx hit	Score	E-value	% ID	TM
c45400_g1	IR25a	KX084508	937	No	BAR64798.1|ionotropic receptor [*Ostrinia furnacalis*]	1709	0.00E + 00	97%	3
c46878_g3	IR1	KX084509	652	Yes	BAR64810.1|ionotropic receptor [*Ostrinia furnacalis*]	1144	0.00E + 00	87%	3
c47407_g1	IR2	KX084510	657	Yes	KOB72397.1|Ionotropic receptor [*Operophtera brumata*]	662	0.00E + 00	53%	4
c43961_g1	IR3	KX084511	659	NO	XP_013194002.1|PREDICTED: glutamate receptor ionotropic, NMDA 2A [*Amyelois transitella*]	1098	0.00E + 00	78%	4
c47528_g1	IR4	KX084512	548	Yes	BAR64809.1|ionotropic receptor [*Ostrinia furnacalis*]	875	0.00E + 00	77%	3
c51268_g2	IR5	KX084513	488	No	BAR64796.1|ionotropic receptor [*Ostrinia furnacalis*]	756	0.00E + 00	74%	0
c45899_g2	IR6	KX084514	499	Yes	BAR64797.1|ionotropic receptor [*Ostrinia furnacalis*]	781	0.00E + 00	85%	4
c46846_g2	IR7	KX084515	578	Yes	BAR64800.1|ionotropic receptor [*Ostrinia furnacalis*]	805	0.00E + 00	71%	3
c49721_g1	IR8	KX084516	371	Yes	BAR64803.1|ionotropic receptor [*Ostrinia furnacalis*]	451	3.00E−150	60%	2
c12165_g1	IR9	KX096209	162	No	KPJ10740.1|Glutamate [NMDA] receptor subunit 1 [*Papilio machaon*]	258	6.00E−82	98%	1
c43148_g1	IR10	KX096210	133	No	ADR64688.1|putative chemosensory ionotropic receptor IR1 [*Spodoptera littoralis*]	153	8.00E−41	62%	1
c37515_g1	GR1	KX084517	372	No	NP_001233217.1|gustatory receptor 68 [*Bombyx mori*]	194	6.00E−54	37%	8
c42471_g1	GR2	KX084518	333	Yes	NP_001233217.1|gustatory receptor 68 [*Bombyx mori*]	148	4.00E−35	34%	6
c38597_g1	GR3	KX084519	221	No	AGA04648.1|gustatory receptor [*Helicoverpa armigera*]	224	2.00E−66	74%	4
c40152_g1	GR4	KX084520	454	Yes	AGK90023.1|gustatory receptor 1 [*Helicoverpa assulta*]	622	0.00E + 00	72%	6
c45098_g1	GR5	KX084521	427	Yes	AGK90012.1|gustatory receptor 5 [*Helicoverpa armigera*]	444	2.00E−149	53%	6
c30207_g1	GR6	KX096211	156	No	XP_013189983.1|PREDICTED: gustatory receptor for sugar taste 64f-like [*Amyelois transitella*]	214	8.00E−64	73%	3
c16511_g1	GR7	KX096212	170	No	KOB74472.1|Gustatory receptor 53 [Operophtera brumata]	230	2.00E−70	71%	3
c127_g1	GR8	KX096213	147	No	DAA06380.1|TPA: gustatory receptor 17 [*Bombyx mori*]	113	8.00E−27	38%	3
c21792_g1	GR9	KX096214	122	No	XP_013189983.1|PREDICTED: gustatory receptor for sugar taste 64f-like [*Amyelois transitella*]	122	5.00E−30	54%	3
c15265_g1	GR10	KX096215	117	No	XP_013189983.1|PREDICTED: gustatory receptor for sugar taste 64f-like [*Amyelois transitella*]	153	2.00E−41	48%	3
