# Supplementary material for: Identification of putative chemosensory receptor genes from yellow peach moth Conogethes punctiferalis (Guenée) antennae transcriptome
Source: Sci Rep. 2016 Sep 23;6:32636. doi: 10.1038/srep32636 (PMC5034240; doi:10.1038/srep32636)
Supplement: Supplementary Information [file srep32636-s1.pdf]

Identification of putative chemosensory receptor genes from yellow peach moth *Conogethes punctiferalis* (Guenée) antennae transcriptome

Xing Ge, Tiantao Zhang*, Zhenying Wang*, Kanglai He, Shuxiong Bai

State Key Laboratory for Biology of Plant Disease and Insect Pest, Institute of Plant Protection, Chinese Academy of Agricultural Science, Beijing 100193,China

Table S1 Primer sequences for qRT-PCR.

Gene names	Primer sequences (5'-3')	
	Forward	Reverse
CpunOR1	GGTCCGATGCTCTGTCT	GTTGCTGGTGTCCATGTAT
CpunOrco/ OR2	TGGCGATTGCTAACCTGAT	GAAGTCTTGCTGCGTGGAG
CpunOR3	TCACGTAGTCGGTCACAT	GGCACCGCAGACTAGAT
CpunOR4	GTCATATCGCTGCCCTT	GTGCACTCCAACAGCATG
CpunOR5	GCTCGATACGGACCTCTT	CACTCCCAAGGTATGTCAT
CpunOR7	TCTTGCAGCCCTTCAGTT	TCGACCAGGAGCAGACT
CpunOR8	GACTACGGACGCTTTGCT	TACTCGTGCTCATTTGCTC
CpunOR9	GGCAGAGTGAGACATTCAGT	TGAAACCAGAGGACGGAC
CpunOR10	TCAGGCCGATGACGAAT	TATGCAGCCATGACAATCTT
CpunOR11	CTTCGGCTCAAATGCTGT	GGTTTCTGCGCTTGTCTT
CpunOR12	TCTTGGGTGCCCTTTGAT	GTGCCGAGCATTTGTCT
CpunOR13	CTCACTACCGAGTACCTCGT	GTTGCCACAGTGAGCTTG
CpunOR14	CTGCGAGCATCAACACC	GCCCAGAAACAATAACAAC
CpunOR15	TACCCGCAACAGCAGATT	CACGAGGATTCGGAGACT
CpunOR16	GGGACGAGGCTCAAAGAC	CAGTATGAACTCAGCAGCAAT
CpunOR19	CTGGGCTAACAGATGACC	GTCAATGGTAGCATGAGCT
CpunOR21	TGCCCTTCTGGACCTT	TCGCCTCATAGTCGCCT
CpunOR22	TCCTGTCGCAGCATTACCT	AGCCTTGTGGAGGTTCAT
CpunOR23	TTGCTTCTGCCGTTTCTT	GTATGCCAATGTGGGTGC
CpunOR24	TGCTATAGAAGCGGTTGT	TGTTACAAGCCGTATTGAT
CpunOR25	TCTTCAGCACCAGTATCAGG	AACGCCTTGTCGTCTATT
CpunOR26	CGTCTCCAAGCAGGTCTC	AACAGCCGCATCAGGTAG
CpunOR27	GCAGGAAATAGCAGAATCT	TATGAAATAACGGCACAAC
CpunOR28	CTATGGAACAACGGAGAAAT	CACGAAGACAGCGAAGAT
CpunOR29	TTTTCGCCGTATCACCTT	TGTCTTCCCTTGCCTCCT
CpunOR30	CCAACTGGTTTCCCTTC	CTCTGGATTCTGACGCAC
CpunOR31	TCCCTTCGGCTGACTCT	TCCACCTTCCTCCACAC
CpunOR32	TGCCCTGAACATACTGCC	CCACCAATAGCGGACTG

CpunOR33	CTTCGCATTGTCACCTTC	GCTGGCATATCACATTTAG
CpunOR35	CCAGCGGCGAGTACACAC	CCGACCAAACATAACACCAT
CpunOR36	CAAGCGTTTGGAACAGTTAT	CGTTGCACAGGAATAAGCCCAAT
CpunOR38	CACCACGGTAATCACAGC	GAATGCAGCCTAATCCCT
CpunOR39	TGGCGACTTGTTTGGT	CTCGTGGCGTTAGAGGT
CpunOR40	CACTTGGGCTCTATTTGG	CCTTCGTATCACCGTCTC
CpunOR41	GAGAATGACATCGAGGAG	ACGACGCCGCAATGAGT
CpunOR42	CTGTGGCGAACCGTCTAT	ATCACCCTTTCACGCTCT
CpunOR44	GGTCACCATTGCCTCTTAC	GCTTGCTGCGACAGGATT
CpunOR45	ACCGAAATCTTCCACTCC	TATGATGAACGCAGCACC
CpunOR46	TGGTACTATGCGTTGGT	CATCTCTGCATGATCAGTAT
CpunOR47	GTCGCAGTGGTGTTCATT	GTCCCTTGCTATTCTTGG
CpunOR48	CTACTCCCAAGCGACAAT	CCACCAAACATGAAGATAT
CpunOR49	GTACAGGACGCCCAACTAT	CAGGCCCTCGAATATCAC
CpunOR50	TATGAATCGCATCCCAAC	GCAGCAGCAAGATCTTTAGT
CpunOR51	ACTTGTTTCCTGTGGGTC	TTGATAACGCTGTTGCTT
CpunOR52	TTCACAGCCACGGTAGTAG	GTCCAATCGGCGAAGTAT
CpunOR53	GTTACTTCTATCGTGTTCCCT	TACCGCGTTTATGCAGT
CpunOR55	CTGGAACCGATGTATGAGT	GATGGCGTTGGTAGTGTCT
CpunOR56	TCGTTGGATCAGGGTGTT	TTCATTTGTCATCGGCTT
CpunOR58	TGCGATTCGCTGGTAACT	TCAGATGTCGGCCGGACAT
CpunOR62	TGAGCGAAAGATGATGTAT	CAGAAGCCAAGTCTGAAT
